# Invasive Ponto-Caspian Amphipods and Fish Increase the Distribution Range of the Acanthocephalan *Pomphorhynchus tereticollis* in the River Rhine

**DOI:** 10.1371/journal.pone.0053218

**Published:** 2012-12-31

**Authors:** Sebastian Emde, Sonja Rueckert, Harry W. Palm, Sven Klimpel

**Affiliations:** 1 Biodiversity and Climate Research Centre (BiK-F), Medical Biodiversity and Parasitology, Senckenberg Gesellschaft für Naturforschung (SGN), Goethe-University (GO), Institute for Ecology, Evolution and Diversity, Frankfurt am Main, Germany; 2 School of Life, Sport and Social Sciences, Edinburgh Napier University, Edinburgh, United Kingdom; 3 Aquaculture and Sea-Ranching, Faculty of Agricultural and Environmental Sciences, University of Rostock, Rostock, Germany; University of Otago, New Zealand

## Abstract

Non-indigenous species that become invasive are one of the main drivers of biodiversity loss worldwide. In various freshwater systems in Europe, populations of native amphipods and fish are progressively displaced by highly adaptive non-indigenous species that can perform explosive range extensions. A total of 40 Ponto-Caspian round gobies *Neogobius melanostomus* from the Rhine River near Düsseldorf, North Rhine-Westphalia, Germany, were examined for metazoan parasites and feeding ecology. Three metazoan parasite species were found: two Nematoda and one Acanthocephala. The two Nematoda, *Raphidascaris acus* and *Paracuaria adunca*, had a low prevalence of 2.5%. The Acanthocephala, *Pomphorhynchus tereticollis,* was the predominant parasite species, reaching a level of 90.0% prevalence in the larval stage, correlated with fish size. In addition, four invasive amphipod species, *Corophium curvispinum* (435 specimens), *Dikerogammarus villosus* (5,454), *Echinogammarus trichiatus* (2,695) and *Orchestia cavimana* (1,448) were trapped at the sampling site. Only *D. villosus* was infected with *P. tereticollis* at a prevalence of 0.04%. The invasive goby *N. melanostomus* mainly preys on these non-indigenous amphipods, and may have replaced native amphipods in the transmission of *P. tereticollis* into the vertebrate paratenic host. This study gives insight into a potential parasite-host system that consists mainly of invasive species, such as the Ponto-Caspian fish and amphipods in the Rhine. We discuss prospective distribution and migration pathways of non-indigenous vertebrate (round goby) and invertebrates (amphipods) under special consideration of parasite dispersal.

## Introduction

Globalization, the transfer and invasion of non-indigenous species, has caused widespread biotic homogenization and the replacement of local species [1], resulting in a worldwide biodiversity loss (e.g., [2]). Several mechanisms, such as different environmental tolerance, higher reproduction rates, or different aggression and mutual predation can be involved in the regulation of the competitive interactions between native and non-indigenous species [3]–[5]. Following the replacement of the native fauna, non-indigenous species can transform habitats and even threaten entire ecosystems. They can alter ecosystem processes, causing serious problems to the environment and major economic losses (e.g., [6], [7]).

The invasion of a new habitat by a host species infected with parasites can have different effects on the local parasite fauna: 1) loss of the original parasite burden of the invader (enemy release hypothesis) [8], [9], 2) introduction of new parasite species with the invader (parasite spillover) [10], 3) invasive species can successfully act as intermediate hosts or vectors for existing parasites or diseases (parasite spillback) [10], 4) loss of local parasite species, if the invader replaces local host species, but can not act as intermediate or definitive host in the parasite life cycles (dilution effect) [11], [12].

Ballast water transport has been a main source of unintentional species release in aquatic systems (e.g., [13]), therefore, the ports in the Rhine delta are becoming important gateways for non-indigenous species. More recently, the expansion of navigation routes across river basin boundaries has led to the construction of navigation canals that connect the river Rhine with previously isolated catchments of the Caspian-, Azov-, Black-, Mediterranean-, Baltic-, North-Sea and the Atlantic Ocean [14]–[16]. These new waterways have opened long distance dispersal routes for aquatic species from several bio-geographic areas, where they can spread directly via natural migration or indirectly via ballast water release [15], [17]–[19]. In the period of 1850–2006, a total number of 141 aquatic invasive species were reported in German waters and particularly in the last century the number has risen extensively in freshwater systems [20]. More than two thirds of these non-indigenous species were not only migratory, but were able to establish themselves and to form self-sustaining populations [20]. Some amphipods (e.g. *D. villosus*) and gobiid fish species (e.g. *N. melanostomus*) are typical invaders into Central Europe including Germany [20]–[22]. They originate in the Ponto-Caspian basin and have spread very quickly to European countries via the so-called “central and southern corridor”, the river Rhine (central) and the Main-Danube Channel (southern) in Germany, respectively [17].

Freshwater fishes are known to harbour a variety of different parasite species that often utilize amphipods as first intermediate hosts within their life cycles (e.g., [23], [24]). The parasite fauna of the invasive round goby *N. melanostomus* from the Ponto-Caspian region has been relatively well studied in its native habitats (e.g., [25]). In addition several studies have focused on the parasite composition within invasive habitats, including the upper Danube River basin, the Baltic Sea and the “Great Lakes” USA [25]–[27]. Studies from the Rhine and adjacent river systems are still missing. Comparative studies have shown that non-indigenous gobies tend to loose their native parasite fauna and acquire the generalist parasites from the local fauna in the invaded area, e.g. in the Gulf of Gdańsk, where twelve metazoan parasite species could be detected, 50% of them were typical for the resident gobiids while seven species of the fauna were also found in their native habitat. At this site they were able to take over the roles as definitive, second intermediate, and paratenic host for different parasite species [25]. In the USA (St. Clair River, Lake St. Clair) complete new host-parasite interactions could be described for *N. melanostomus*, with only four out of ten detected parasite species known from their native habitats [28].

Introduced fish species are able to modify native host-parasite dynamics by either increasing or decreasing the parasite burden of native hosts [12]. In the case of the acanthocephalan *Acanthocephalus galaxii,* the introduced brown trout (*Salmo trutta fario)* reduces the parasite burden of the native roundhead galaxias (*Galaxias anomalus*). Even though this appears to be of less concern, it could still have flow-on effects to native species dynamics [12].

Aquatic acanthocephalans use benthic crustaceans (e.g. amphipods) as intermediate hosts, and several studies on the interactions between acanthocephalan parasites and their intermediate hosts exist [24], [29]. However, the function of invasive species as obligatory hosts for non-indigenous acanthocephalans has received only little attention and existing studies report contradicting results so far. Dunn and Dick (1998) [30] observed that the prevalence of a bird acanthocephalan, *Polymorphus minutus*, was higher in the native amphipod *Gammarus duebeni celticus* than in the invader species *Gammarus tigrinus* in a freshwater environment in Ireland. In the same country MacNeil et al. (2003) [31] demonstrated that the fish acanthocephalan *Echinorynchus truttae* is more prevalent in the invasive amphipod *G. pulex* than in the native *G. duebeni celticus* and that acanthocephalan parasites mediate predation between their intermediate macroinvertebrate hosts by lowering the intraguild predation upon the non-infected native form. This leads to species co-existence of the amphipods, or at least slows down species replacement of *G. duebeni celticus* in this particular biological invasion event [31].

**Figure 1:**
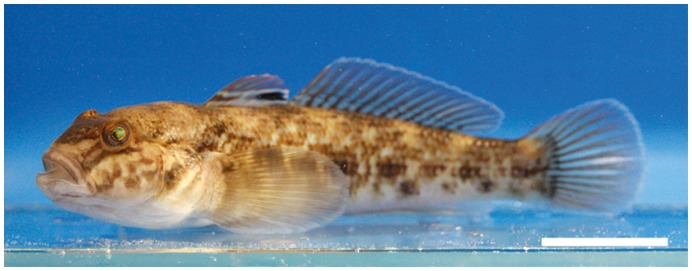
*Neogobius melanostomus.* The investigated goby *Neogobius melanostomus*. Scale bar = 2 cm.

**Figure 2:**
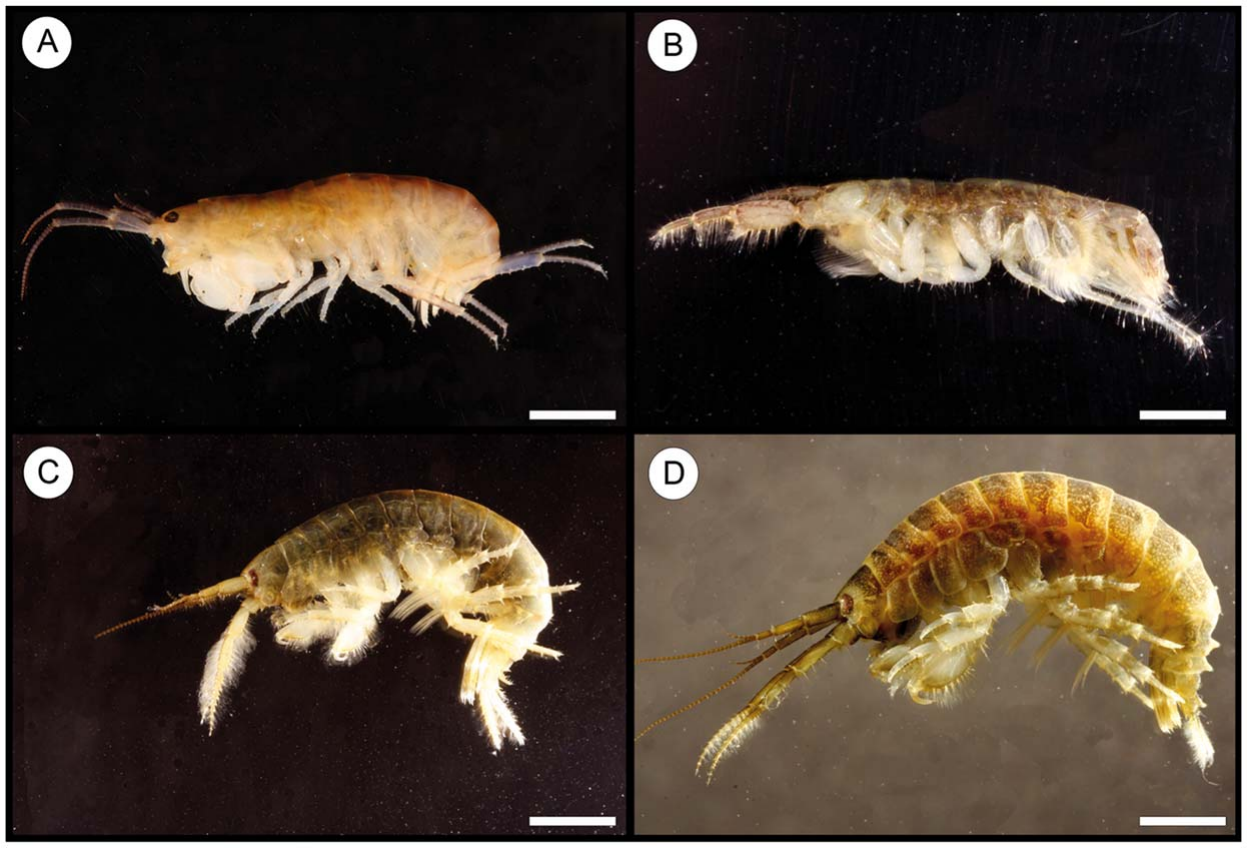
Invasive amphipod species. A) *Orchestia cavimana*. B) *Corophium curvispinum*. C) *Echinogammarus trichiatus*. D) *Dikerogammarus villosus*. Scale bar = 2 mm.

The present study analyses the parasite fauna and feeding ecology of one of the most abundant fish species in the river Rhine near the port of Düsseldorf (Germany), the invasive *N. melanostomus* (Figure 1). The local amphipod fauna, the main food source for *N. melanostomus*, comprising the four invasive species *C. curvispinum*, *D. villosus, E. trichiatus* and *O. cavimana* (Figure 2) were investigated as potential first intermediate hosts for metazoan fish parasites. This study sheds light on the potential role of invasive Ponto-Caspian fish and amphipods on the distribution of the non-indigenous acanthocephalan *P. tereticollis* in the Rhine.

## Results

### Fish Data

#### Biology

*Neogobius melanostomus* (n = 40) had a mean total length of 9.0 cm (range 6.5–13.0 cm) and a mean total weight of 11.3 g (range 3.43–32.27 g). In four juveniles we could not determine the sex, while the other 36 specimens had a balanced sex ratio (50%).

#### Stomach contents

Food components were detected in 37 of 40 examined fish digestive tracts. Beside a small proportion of plant material, we distinguished seven different prey organisms, which belonged to Mollusca, Crustacea and Insecta (Table 1). Crustacea were the main diet component (N = 60.05%, IRI = 12,617), mainly consisting of amphipods (Amphipoda indet. and *D. villosus*; N = 59.59%, IRI = 12,189). Amphipoda indet. contains specimens that could not be confidently identified due to a progressive degree of digestion or fragmentation. Amphipoda indet. consists mainly of *D. villosus*, and to a much lesser extent of *E. trichiatus,* which co-exists with *D. villosus,* but was not identified among the non-digested specimens of the stomach content. Because of the small body size of *C. curvispinum,* this species can be excluded from the pool of species that could comprise Amphipoda indet. *Orchestia cavimana* can also be excluded as it lives away from the gobies feeding range, at and above the water surface at the riverbank. The IRI’s of Insecta (IRI = 1,496) and Mollusca (IRI = 1,723) were similar, but only about one tenth as important as the IRI of the Crustacea. The insect suborder Nematocera indet. (N = 23.11%, IRI = 853) and the mollusc species *Sphaerium corneum* (N = 4.12%, IRI = 790) represented two prey organisms with the highest relative importance within their respective groups.

**Table 1:** Feeding ecology of *Neogobius melanostomus*.

Prey organism	n	F [%]	W [%]	N [%]	IRI
**Mollusca**	**47**	**56.76**	**19.64**	**10.73**	**1,723.68**
*Ancylus fluviatilis*	29	29.73	4.28	6.64	324.47
*Sphaerium corneum*	18	40.54	15.39	4.12	790.96
**Crustacea**	**263**	**91.89**	**77.26**	**60.05**	**12,616.98**
*Dikerogammarus villosus*	72	21.62	25.89	16.48	915.97
*Corophium curvispinum*	1	2.70	0.09	0.12	0.56
Amphipoda indet.	189	67.57	51.31	43.25	6,389.29
*Asellus aquaticus*	1	2.70	0.02	0.23	0.68
**Insecta**	**127**	**48.65**	**1.76**	**29.00**	**1,496.28**
Nematocera indet. (Larve)	101	35.14	1.19	23.11	853.88
Trichoptera indet. (Larve)	26	16.22	0.57	5.95	105.78
**Plantae**	**1**	**2.70**	**1.34**	**0.23**	**4.25**
Plantae indet.	1	2.70	1.34	0.23	4.25

F = „frequency of occurrence“, IRI = „index of relative importance“, N = „numerical percentage of prey“, n = „number of prey organisms” and W = „weight percentage of prey“.

#### Parasite fauna

**Figure 3:**
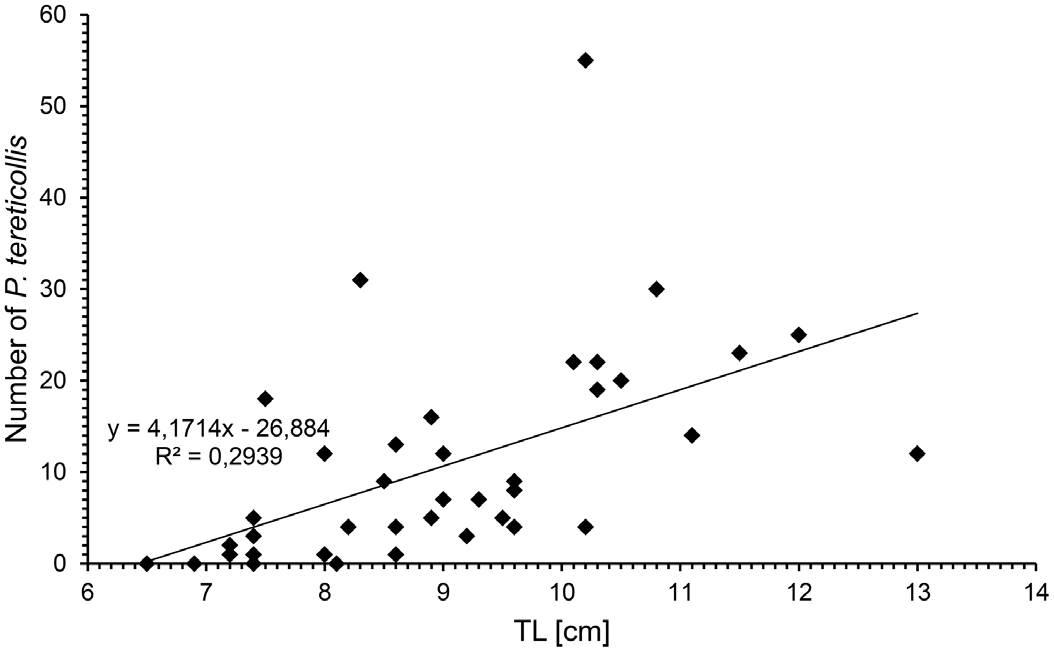
Number of isolated *Pomphorhynchus tereticollis* in relation to the total length [TL] of *Neogobius melanostomus.* A significant relation between the number of *Pomphorhynchus tereticollis* specimens and the total length of *Neogobius melanostomus* was detected. With increasing size of the fish the intensity of parasites increases.

Three metazoan parasite species were found (Table 2). *Raphidascaris acus* and *P. adunca*, both Nematoda, had a low prevalence of 2.5%. The acanthocephalan *P. tereticollis* was the predominant parasite species (in total P = 90.0%, mA = 10.7). This parasite occurred in the cystacanth stage only (75.0% encysted in the mesenteries and liver and 25% free in the body cavity). The data (n = 40) show a significant correlation between total length of *N. melanostomus* and the respective intensity of infection (Spearman’s rank test; r = 0.70, p<0.0001; R^2^ = 0.29) (Figure 3).

**Table 2:** Parasitological calculations of the parasite fauna of *Neogobius melanostomus*.

Parasite species	Stage	Organ	P [%]	I	mI	mA
**Nematoda**						
*Raphidascaris acus*	l	BC	2.5	2	2.0	0.05
*Paracuaria adunca*	l	In	2.5	1	1.0	0.03
**Acanthocephala**						
*Pomphorhynchus tereticollis*	l	L/Mes	90.0	1–55	11.9	10.70

In = intestine, I = Intensity, l = larvae, L = liver, BC = body cavity, mA = mean abundance, Mes = mesentery, mI = mean intensity and P = prevalence.

### Amphipod Data

#### Biology

No endemic amphipods were found at the sampling locality. In total, 10,032 amphipods were collected; *C. curvispinum* (4.34%), *D. villosus* (54.37%), *E. trichiatus* (26.86%) and *O. cavimana* (14.43%) (Figure 2). *Corophium curvispinum* represented the smallest species with an average total length of 4.3 mm (1.0–7.0 mm) and an average total weight of 0.003 g (0.001–0.007 g). The other three species had an average total length of 10.1 mm to 12.2 mm with a range of 5.0–18.0 mm. *Dikerogammarus villosus* was the largest species with an average total weight of 0.045 g (0.004–0.108 g).

#### Parasite fauna

**Figure 4:**
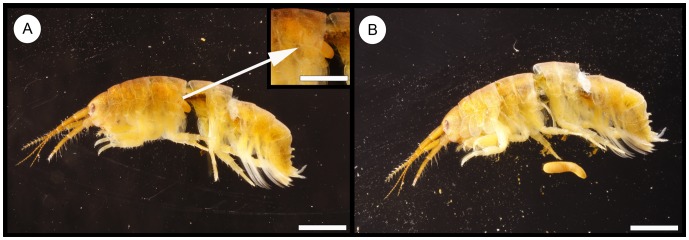
Infected amphipod. *Dikerogammarus villosus* as intermediate host for *Pomphorhynchus tereticollis*. A) Larval stage (late Acanthella) located in the body cavity. B) Isolated Acanthella larvae. Scale bar = 2 mm.

**Figure 5:**
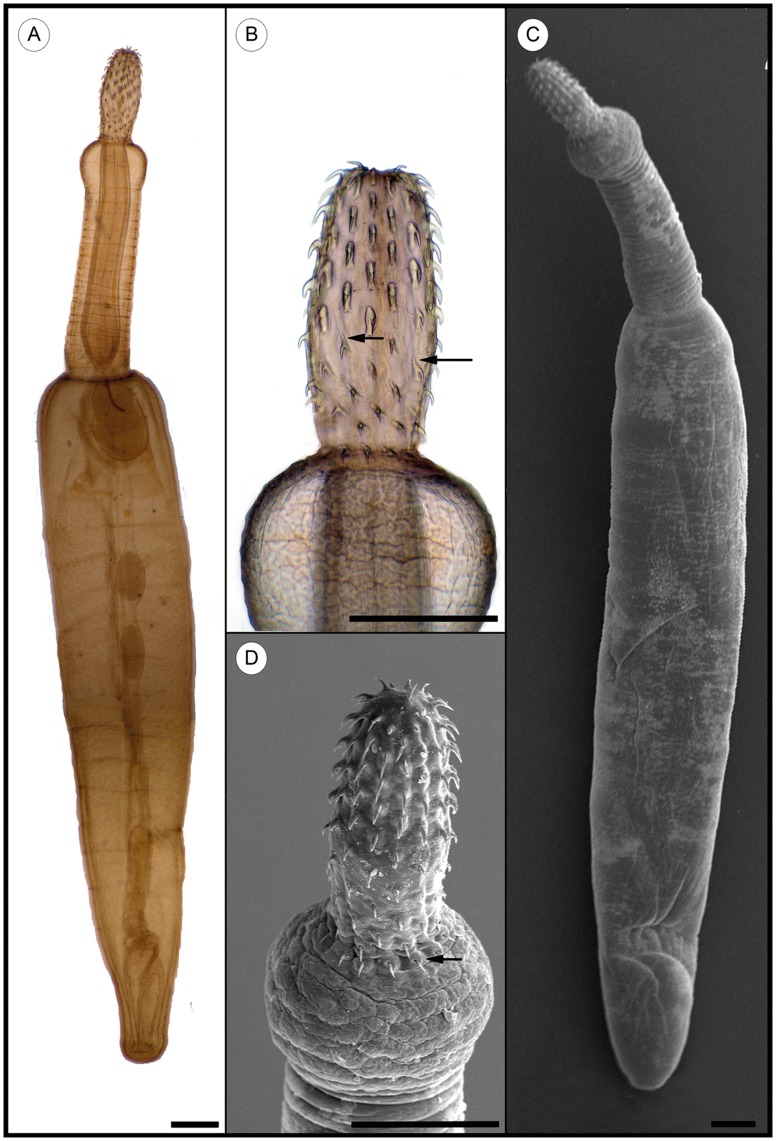
Larval stage (Cystacanth) of the fish parasite *Pomphorhynchus tereticollis* isolated from the paratenic host *Neogobius melanostomus*. A+C) Habitus of *Pomphorhynchus tereticollis*, light- and scanning electron microscopy. B+D) Detail of proboscis. Number and species specific structure details (arrows) of the proboscis hooks are clearly visible. Scale bar = 500 µm.

Parasites were detected exclusively in *D.* *villosus*. Two out of 5,454 amphipods were infected with acanthocephalan larval stages (Figure 4), the Acanthella larvae (P = 0.04%, I = 1, mI = 1.00, mA = 0.0004). The isolated larvae were both identified as *P. tereticollis* (Figure 5).

## Discussion

### Parasite Fauna of *Neogobius Melanostomus*

A wide variety of parasites have been recorded in this non-indigenous fish species including 35 metazoan species in Europe (e.g., [25]–[27], [32]). In its native habitats *N. melanostomus* usually carries more than 10 different parasite species. Machevsky et al. (1990) [33] and Kvach (2005) [34] both reported 16 species for the Black Sea. Only three parasites are present at this study site (Table 2). Therefore, we can assume that the invasive fish has lost the majority of its native parasite fauna, which would support the enemy release hypothesis. Apart from the most abundant parasite species *P. tereticollis* (Acanthocephala, P = 90.0%), the nematodes *R. acus* and *P. adunca* were isolated at a low prevalence of 2.5% respectively. *Raphidascaris acus* has been previously isolated at higher prevalences from non-indigenous gobies from the Danube and Rhine rivers (57% and 56% respectively) [26], [35], while *P. adunca* has been recorded at a similar prevalence (2.1%) from gobies in the Baltic Sea (Kiel Canal) only [36]. The main final hosts for *R. acus* are pike (*Esox lucius*) and brown trout (*S. trutta fario*) [37], whose stocks are rather low in the Rhine River [38], which may explain the low prevalence rates. *Paracuaria adunca* is distributed worldwide and has a three-host life cycle [37]. The first intermediate hosts are various species of amphipods, and the Rhine could be an ideal habitat, with its rich amphipod fauna, given that an appropriate host type is available [37], [39]. Fishes such as *N. melanostomus*, which apparently serve as second intermediate hosts in the river Rhine, get infected by oral intake of the first intermediate hosts. Specified final hosts are bird species of the family Laridae [40]. The sporadic occurrence of gulls explains the low prevalence (P = 2.5%) of *P. adunca* in *N. melanostomus*. Due to their low infestation rates, both nematodes do not play an important ecological role at this sampling site. While *P. adunca* was recorded only twice in previous goby studies, *R. acus* is usually a common parasite with high prevalence [26], [35].

In our study species, myxozoans, digeneans, monogeneans and cestodes were absent. Some monogeneans (e.g. *Dactylogyrus* spp.), digeneans (*Diplostomum spathaceum*, *Rhipidocotyle* spp., *Tylodelphis clavata*) and cestode species (e.g. *Proteocephalus* spp.) were absent from *N. melanostomus*, even though they have been reported from this goby species in other locations [25], [26], [34], [41] and were also detected in sympatric roach (*R. rutilus*) and perch (*P. fluviatilis*) at the same time [42]. The most likely reason is the recent invasion history, but further research is needed to explain why the gobies were not infected with parasite species that are present at this sampling site and already known to infest *N. melanostomus*. Kvach & Skora (2007) [25] give a nice example how the parasite fauna of the invasive goby *N. melanostomus* in the Gulf of Gdansk increases from 1–4 to 5–12 parasite species over a time period of two years. With its recent invasion history, it is expected that the invasive goby will acquire more parasites over time including the earlier mentioned local species.

### Fish Biological Data and Feeding Ecology

The maximum size of *N. melanostomus* in this study was 13.0 cm. Size differs in various habitats and it seems that these gobies tend to reach larger sizes in brackish than in fresh waters [43]. The diet of the goby consists of various prey organisms at this sampling site, but is dominated by crustaceans (IRI = 12,617), particularly amphipods (IRI = 12,596; Table 1). The high abundance of amphipods in the intestinal tract of *N. melanostomus* indicates a large population size of amphipods in the Rhine River. Amphipods and gobies are both benthic organisms, but the high biomass of amphipods at this sampling site is probably the main reason for the preference of these prey items. This is supported by the study of Rakauskas et al. (2008) [44], in which the more abundant *Dreissena polymorpha* (Bivalvia) was fed on rather than amphipods. There is a link between the parasite *P. tereticollis* and the amphipod *D. villosus* that is used as first intermediate host. The acanthocephalan occurred with a high prevalence of 90.0% and an average intensity of 11.9 individuals per goby. Considering the low prevalence of *P. tereticollis* in the amphipods (P = 0.04%), the high infection rate of *P. tereticollis* in the gobies seems unusual. However, acanthocephalans are known to manipulate their host’s behavior in order to facilitate transmission to the final host. In contrast to non-infected hiding amphipods, infected amphipods become photophilic, move to the free water column and are more likely to be preyed upon [24]. Additionally, the host’s condition is lowered indirectly by the acanthocephalans, making infected amphipods more attractive for predators. Therefore, *N. melanostomus* doesn’t need to consume vast numbers of amphipods to acquire this parasite. Paterson et al. (2011) [12] reported comparable data with a maximum of only 0.1% of collected amphipods being infected by the studied acanthocephalan. This is also supported by Busch et al. (2012) [45] who describe and discuss low infection rates of different crustaceans, which predominantly act as first intermediate hosts for aquatic parasites. Further sampling in different seasons as well as laboratory experiments would be required to describe the dietary preferences of the gobies in more detail in future.

### Amphipoda Fauna

The presence of exclusively non-indigenous species at the sampling site confirms the statement by Eggers and Martens (2001) [46] that the amphipod fauna, especially in larger rivers like the Rhine, is particularly affected by invasive species. While *O.* *cavimana* originates from the eastern Mediterranean Sea, *C.* *curvispinum*, *D. villosus* and *E. trichiatus* originate in the Ponto-Caspian region (Black Sea, Caspian Sea). Parasite larvae were only detected in *D. villosus*. Two out of 5,454 analyzed individuals were infested with larval stages (Acanthella) of the acanthocephalan *P. tereticollis* (P = 0.04%). It seems as if this parasite has successfully integrated the non-indigenous intermediate host *D. villosus* in its life cycle. Such a process is known as lateral incorporation [47]. This adaptation demonstrates that *P. tereticollis* is a cosmopolitan generalist. The common intermediate host *Gammarus pulex* [48] was not detected in the stomach analyses of *N. melanostomus* or in the macrozoobenthos samples. The high infection rate of *N. melanostomus* with *P. tereticollis* suggests that the parasite must have performed a host switch of the obligate first intermediate host from *G. pulex* to *D. villosus*.

### *Pomphorhynchus tereticollis* Distribution and Migration Ways

In the present study, mainly the cystacanth larvae of the Acanthocephala *P. tereticollis* were isolated from the mesenteries and liver of *N. melanostomus* with a prevalence of 90.0%, and are therefore of ecological importance in this system. As amphipods usually act as obligate first intermediate hosts, *N. melanostomus* could be used as paratenic host in the life cycle of *P. tereticollis*. Adult parasites are most likely to be found in chub (*Leuciscus cephalus*) and barbel (*Barbus barbus*), which still has to be proven in this area. Although these final hosts are not known as primarily piscivorous, we suppose that especially larger barbels feed on these gobies, as they regularly feed on smaller fish species. Hine and Kennedy (1974) [49] described that the closely related parasite *Pomphorhynchus laevis* occasionally matures in trout (*Salmo trutta*), and also catfish (*Silurus glanis*) [50] harbors this parasite and should be considered as a possible final host and re-examined in this regard. Hence, we expect to find mature *P. tereticollis* in barbels, trout and maybe in European catfish in this area. Alternatively, *N. melanostomus* may act as a dead-end host for this acanthocephalan. If a suitable final host for the parasite does not consume the infected gobies, the life cycle gets interrupted, which would result in a continued loss of infection within the system. This would conform to the dilution effect, which has been described for different parasite-host systems [11], [51]. To reject or accept this hypothesis, further investigations of prevalence and intensities of *P. tereticollis* in different fish hosts (final as well as the paratenic hosts) should be conducted. The data would have to show a decrease of the parasite’s occurrence in order to confirm the dilution effect. As *N. melanostomus* has reached an enormous population size in the Rhine we would expect this to become apparent rather quickly.

So far, *P. tereticollis* was documented only in the fish host flounder (*Platichthys flesus*) from the German Baltic Coast [52], but in this study we were able to report its presence in *N. melanostomus* for the first time in German inland waters. *Pomphorhynchus tereticollis* was treated as a synonym for *P. laevis* for a long time [52]–[54]. Furthermore, morphological similarities and a similar host spectrum of *P. tereticollis* and *P. laevis* may have led to incorrect identifications in the past. Studies on the parasite fauna of *N. melanostomus* were carried out across Europe, but only a few studies exist from Germany. Kvach and Winkler (2011) [29] studied the parasite fauna of *N. melanostomus* in brackish waters from the German coast of the Baltic Sea. Nachev et al. (2010) [35] investigated *N. melanostomus* from the river Rhine in Germany (near Grieth, Kalkar city at Rhine kilometer 844), which makes the study the most suitable for comparison.

In previous studies, only the closely related species *P. laevis* was identified in *N. melanostomus* (e.g., [25], [32], [55]). Molnár (2006) [56] detected a similar prevalence for *P. laevis* in *N. melanostomus* (P = 93.0%) in the Danube river. Nachev et al. (2010) [35] showed almost undistinguishable data in terms of prevalence (91.2%), mean intensity (11.1), intensity (1–44) and mean abundance (10.15) although for *P. laevis* and not *P. tereticollis* (compare Table 2). Due to its continued misidentification and synonymy of *P. tereticollis* and *P. laevis* it is important to state here clearly that the isolated acanthocephalans in this study were confidently identified as *P.* *tereticollis* based on well-defined, but often overlooked morphological differences to *P. laevis*. Three morphological different proboscis hooks can be recognized in the present material, which can be used to distinguish between both species. The hooks on the posterior proboscis half of *P. tereticollis* have developed an anterior extension of the base in contrast to *P. laevis* (Figure 5b). The final hooks, located most posterior on the proboscis on the top of the bulbus, are another typical character of *P. tereticollis* (Figure 5d). The middle hooks of *P. tereticollis* compared to the surrounding hooks are significantly thicker, while the hooks of *P. laevis* have nearly the same size (Figure 5b) [52], [56]. Our data and Nachev et al. (2010) [35] demonstrate that all detected parasite species occurred exclusively in the larval stage, indicating that *N.* *melanostomus* acts as an intermediate host in the river Rhine. Because *N. melanostomus* has only a recent history of invasion, it appears that no parasite species has yet been able to use it as a definitive host. This supports the “enemy release hypothesis” and gives comparable results to the study of Kvach and Stepien (2008) [27], who documented only one adult parasite species and described a consistent low parasite load for the great Lakes (USA) in the last decade in comparison to their native habitats. The loss of native parasites and acquiring only a few local generalist parasite species result in a higher fitness and might be one of the main drivers of the success of this invader.

Furthermore, a significant relation between the number of *P.* *tereticollis* specimens and the total length of *N. melanostomus* was seen. With increasing fish size the number of parasites increases (Figure 3). There are different possible reasons for this correlation. 1) Larger/older gobies can accumulate more parasites over a longer time period than smaller/younger gobies. 2) Larger/older gobies can feed on larger amphipods, which could be important as the development of *P. tereticollis* larvae might only take place in amphipods above a certain minimum size. The fact that all four parasite free fishes were small (6.5–8.1 cm) points in that direction. 3) A change of dietary preferences of the fish towards amphipods after reaching a certain fish length. The third reason is the least likely, because of the large number of smaller amphipods in the stomach contents of the small gobies.

The North and Baltic Sea are described as the native habitats and the fish families Acipenseridae, Gadidae and Salmonidae as the final hosts of *P. tereticollis* [56]. Recently this parasite started to occur in Europe in fish (e.g. *L. cephalus*) and amphipod (e.g. *G. pulex*) hosts in France and Slovakia [52], [54], [57]. A possible explanation for the introduction of *P. tereticollis* into German inland waters is the invasion of *D. villosus* and *N. melanostomus* through the “southern corridor” [20], including the Danube River, which passes Slovakia where *P. tereticollis* occurs. It is possible that the invaders become infected with *P. tereticollis* while passing through Slovakia, and subsequently distribute the parasite towards Germany. Another explanation is that *P. tereticollis* has been introduced to the German river systems through the appropriate final hosts a long time ago, establishing itself most recently after colonization of the rivers with the suitable intermediate hosts such as the invasive *D. villosus* and *N. melanostomus*. In this case *P. tereticollis* can be considered as an invasive species of German inland waters. Some final hosts described by Golvan (1969) [56] such as salmonids are anadromous migratory fish. Therefore, an introduction of the parasite through the tributaries of the North and Baltic Sea, such as the Rhine delta, is a realistic scenario. Another alternative scenario could be the continuous coexistence of *P. tereticollis* and *P. laevis* in these habitats. Misidentification, caused by the morphological similarity of these two *Pomphorhynchus* species, might have led to incorrect distribution records for *P. tereticollis*.

### Conclusion

Non-indigenous species represent new potential hosts for native parasites or possibly introduced parasites and diseases. These events often lead to an elimination of local species like in the present study, as the native amphipods appear to be completely displaced by non-indigenous species at the sampling site (Rhine River near Düsseldorf, North Rhine-Westphalia, Germany). We suspect that these invasive amphipod and gobiid fish species, especially *D. villosus* and *N. melanostomus*, play a decisive role in the life cycle biology and transmission strategy of a putatively introduced parasite, the acanthocephalan *P. tereticollis*. We identified a completely new limnic host-parasite interaction of three non-indigenous organisms, which originated from entirely different localities (marine, fresh and brackish water habitats). On the one hand, the acanthocephalan parasite *P. tereticollis* from the Baltic and North Sea, but also from fresh water habitats (France, Slovakia), and on the other hand *D. villosus* and *N. melanostomus*, two invasive species originating from the Ponto-Caspian region. Both species act as intermediate hosts, the amphipods as a common first obligatory intermediate host and the goby as a paratenic host, which serves to further spread the parasite. We show here, that two invasive species act as intermediate hosts for an almost certainly non-indigenous parasite species, this could contribute to its continuous range expansion, providing that their transmission to the next host is successful. Suitable final hosts in the life cycle of *P. tereticollis* such as barbel (*B. barbus*) and chub (*L. cephalus*) will most likely have to struggle with increasing infections of *P. tereticollis*. This can either lead to a co-existence or a displacement of the previously dominating native acanthocephalan *P. laevis*. Responsible is the change in amphipod fauna, first intermediate hosts for *P. laevis* such as *G. pulex* were displaced by *D. villosus*, the suitable intermediate host for *P. tereticollis*. The burden of the new parasite could increasingly affect the fitness of the vertebrate and invertebrate hosts and should be focused on in further studies.

## Materials and Methods

### Ethics Statement

An approval by a review board institution or ethics committee was not necessary, because all the fish in the current study were self-caught by fishing rod holding a valid local fishing license (No. 900819), issued by the “Rheinfischereigenossenschaft”, 53639 Königswinter, Germany. We confirm that no live fish were used. In Germany, the fishing license permits the holder to capture and sacrifice the fish, which can be used for research purposes. All the fish were stunned by a blow on the head and expertly killed immediately by cervical dislocation and a cardiac stab according to the German Animal Protection Law (§ 4) and the ordinance of slaughter and killing of animals (Tierschlachtverordnung § 13). Because of public accessibility no permissions were required to enter the sampling site.

### Sampling

Fish samples of *N. melanostomus* (n = 40) were collected during May and June 2009 by fishing rod at the Rhine River, North-Rhine Westphalia, Germany (between the ports of Neuss and Düsseldorf, river kilometer 742) and stored in a deep freezer at −20°C. Amphipods (n = 10,032) were sampled within 3 days in June 2009 at the same site by using the “kick-sampling” method after Storey et al. (1991) [58]. During sampling, the amphipods were kept together with organic material and some stones in ten-liter buckets. The entire samples were frozen at −20°C, and later separated from sediment and identified to species in the laboratory.

### Biological Data and Parasitological Examination

Each goby was measured for weight (g) and total length (cm). All specimens were analyzed for their stomach content and metazoan parasite fauna, using a stereomicroscope. Isolated food organisms and parasites were preserved in an alcohol mixture (70% ethanol and 4% glycerol). The amphipods were thawed in a refrigerator, separated under a stereomicroscope from the sediment, identified to species by using the key of Eggers & Martens (2001, 2004) [46], [59] and preserved in 70% ethanol. Fifty amphipods of each species were measured in relation to body size and weight with an ocular micrometer and an analytical balance. The amphipods were measured under a stretched condition from the anterior rostrum to the base of the telson [60]. For the parasitological examination, amphipods were digested in a freshly prepared pepsin hydrochloric acid solution (250 ml aqua dest., 1.75 g pepsin, 1.5 g sodium chloride (NaCl), 1 ml hydrochloric acid (HCl = 37%)) for about six to nine hours [61]. The amphipods were dissected in pieces and carefully examined. The isolated parasites were stored in 70% ethanol and 4% glycerol.

### Morphological Identification

For parasite identification glycerin preparations were made according to Riemann (1988) [62]. A microscope was used to examine and document the parasites. Some specimens were processed for scanning electron microscopy (SEM) [63]. Literature used for parasite identification included original descriptions, as well as descriptions of Golvan (1969) [56] and Ŝpakulovà et al. (2011) [52] for the Acanthocephala, and Moravec (1994) [37] for the nematode species.

### Parasitological Data

The prevalence (P), mean abundance (mA), mean intensity (mI) and intensity (I) were calculated for each parasite species according to Bush et al. (1997) [64].

### Fish Stomach Content Analyses

Since gobies have no clearly demarcated stomach, the entire gastrointestinal tract was examined. Prey organisms were sorted and identified to the lowest possible taxon and grouped into taxonomic categories. The numerical percentage of prey (N%), the weight percentage of prey (W%), and the frequency of occurrence (F%) were determined [65], [66]. On basis of these three indices, the index of relative importance IRI of food items was calculated [67]. Increasing values of N, W, and F generally result in an increased IRI and present a higher importance of a specific prey organism.
